# The Relationship between COVID-19 Severity in Children and Immunoregulatory Gene Polymorphism

**DOI:** 10.3390/v15102093

**Published:** 2023-10-14

**Authors:** Kateryna Kozak, Halyna Pavlyshyn, Oleksandr Kamyshnyi, Oksana Shevchuk, Mykhaylo Korda, Sandor G. Vari

**Affiliations:** 1 Department of Pediatrics No. 2, I. Horbachevsky Ternopil National Medical University, 46001 Ternopil, Ukraine; pavlishin@tdmu.edu.ua; 2 Department of Microbiology, Virology, and Immunology, I. Horbachevsky Ternopil National Medical University, 46001 Ternopil, Ukraine; kamyshnyi_om@tdmu.edu.ua; 3 Department of Pharmacology and Clinical Pharmacology, I. Horbachevsky Ternopil National Medical University, 46001 Ternopil, Ukraine; shevchukoo@tdmu.edu.ua; 4 Department of Medical Biochemistry, I. Horbachevsky Ternopil National Medical University, 46001 Ternopil, Ukraine; korda@tdmu.edu.ua; 5 International Research and Innovation in Medicine Program, Cedars–Sinai Medical Center, Los Angeles, CA 90048, USA; vari@cshs.org

**Keywords:** ACE2, IFNAR2, TYK2, OAS1, OAS3, CD40, FCGR2A, CASP3, COVID-19, children

## Abstract

Coronavirus disease (COVID-19) and its outcomes remain one of the most challenging problems today. COVID-19 in children could be asymptomatic, but can result in a fatal outcome; therefore, predictions of the disease severity are important. The goal was to investigate the human genetic factors that could be associated with COVID-19 severity in children. Single-nucleotide polymorphisms of the following genes were studied: ACE2 (rs2074192), IFNAR2 (rs2236757), TYK2 (rs2304256), OAS1 (rs10774671), OAS3 (rs10735079), CD40 (rs4813003), FCGR2A (rs1801274) and CASP3 (rs113420705). In the case–control study were 30 children with mild or moderate course of the disease; 30 with severe COVID-19 symptoms and multisystem inflammatory syndrome in children (MIS-C) and 15 who were healthy, and who did not have SARS-CoV-2 (PCR negative, Ig G negative). The study revealed that ACE2 rs2074192 (allele T), IFNAR2 rs2236757 (allele A), OAS1 rs10774671 (allele A), CD40 rs4813003 (allele C), CASP3 rs113420705 (allele C) and male sex contribute to severe COVID-19 course and MIS-C in 85.6% of cases. The World Health Organization reported that new SARS-CoV-2 variants may cause previously unseen symptoms in children. Although the study has limitations due to cohort size, the findings can help provide a better understanding of SARS-CoV-2 infection and proactive pediatric patient management.

## 1. Introduction

Coronavirus disease (COVID-19) and its outcomes remain amongst the most pressing problems today. In October 2023, the total count of confirmed COVID-19 cases reached 771 million worldwide. Notwithstanding vaccination, the mortality rate is still high. As of the end of September 2023, 13.5 billion vaccinations had been given against SARS-CoV-2; 66.12% of adults had completed the primary series of vaccinations; 31.74% of the world population received boosters or additional dosages, but from January till October 2023 more than 237 thousand deaths related to COVID-19 were registered [1]. Despite the assumption that COVID-19 in children and adolescents has an asymptomatic course, fatal outcomes are also noticed in the pediatric population. Based on UNICEF Data, during the COVID-19 pandemic (till October 2023) there were 17,490 COVID-19 deaths among children and adolescents—58.75% were registered in the age group 10–19 and 41.25% in children aged 0–9 years [2]. COVID-19 in childhood can also lead to multisystem inflammatory syndrome in children (MIS-C). Data from the USA reported an MIS-C frequency of 316 cases per million confirmed SARS-CoV-2 episodes in persons aged <21 [3]. Recent studies underline that critical illness or even death among children with COVID-19 is most commonly seen in patients with comorbidities (respiratory, cardiovascular disorders, obesity, neurological or oncological disease) and the presence of co-infections [3,4,5]. In addition, age is an important risk factor for disease severity—premature babies, infants and adolescents are at greater risk for poor COVID-19 outcomes [3,5,6].

The course of any viral infection as well as COVID-19 will depend on the characteristics of the pathogen (its structure, viral load and the site of penetration into the host’s cells), the type of host immune response and transmission environment, along with its influencing factors [6,7]. It must be mentioned that external factors (stress, sleep, nutrition and microbiota type) can undergo corrections, but the host genetic factor cannot be corrected or changed, and has a very significant impact on the course of the viral infection [6].

Therefore, the research goal was to investigate human genetic factors that could be associated with COVID-19 severity in the pediatric population. Single-nucleotide polymorphisms (SNPs) of the following genes were studied: ACE2 (rs2074192); IFNAR2 (rs2236757); TYK2 (rs2304256); OAS1 (rs10774671); OAS3 (rs10735079); CD40 (rs4813003); FCGR2A (rs1801274) and CASP3 (rs113420705).

The angiotensin converting enzyme 2 (ACE2) gene has several single-nucleotide polymorphisms that are associated with communicable and noncommunicable diseases. ACE2 alleles are risk factors for cardiovascular diseases (hypertension, coronary artery disease, heart failure, atherosclerosis), respiratory diseases (pulmonary hypertension, chronic obstructive pulmonary disease, asthma, acute lung injury, acute respiratory distress syndrome, lung cancer, pulmonary sarcoidosis) and endocrine diseases (type 2 diabetes mellitus, obesity) [8,9,10]. Since 2019, SARS-CoV-2 infection has become the most commonly discussed communicable disease related to ACE2 gene expression. Recent studies show that intronic SNP rs2074192 (variant g42403) is related to COVID-19 severity as well as concomitant comorbidities [11].

The interferon system is extremely powerful in the context of antiviral defense. The action of the type I interferon is mediated by the interaction with interferon alpha and beta receptor subunit 1 (IFNAR1) and interferon alpha and beta receptor subunit 2 (IFNAR2) receptors. The expression of IFNAR2 is regulated by the corresponding genes. Recent studies suggest the influence of IFNAR2 gene polymorphism on the course and severity of SARS-CoV-2 infection, especially its risk allele A [12,13].

The interferon system is closely related to tyrosine kinase 2 (TYK2). TYK2, as part of the Jak-family, is discussed as one of the key pathogenetic substances in immune-mediated inflammatory disease development [14]. Current knowledge suggests that changes in TYK2 regulation lead to deviations in interferons α and β response. The downregulation of TYK2 with other Jak-family members (Jak1, Jak2, Jak3) is linked to cytokines action—IL-6, IL-10, IL-11, IL-12, IL-19, IL-20, IL-22 and IL-23 [14,15]. The consequences of the TYK2 gene’s (rs2304256) influence on COVID-19 are still controversial. Previous research has shown discordant results. Dieter C. et al. and Benmansour R. with their colleagues demonstrated a tendency in the association of the AA genotype TYK2 gene (rs2304256) with severe and lethal outcomes in adult patients with COVID-19 [16,17]. At the same time, Risi et al. reported an association of the A allele carrier with mild disease course, and underlined its protective properties [18]. Therefore, the influence of genotypes and alleles of the TYK2 gene (rs2304256) remains a point of concern.

Among interferon-induced enzymes, 2′,5′-oligoadenylate synthetase (OAS) must be mentioned, as it has a great role in antiviral immunity. Much research suggests that OAS1 rs10774671 with the risk allele A plays a dominant role in the regulation of OAS enzymatic activity and SARS-CoV-2 elimination [19]. Single nucleotide variant rs10735079 (A > G) is responsible for gene clusters OAS1, OAS2 and OAS3, and is among the COVID-19 genes [12,20]. Studies in the adult population suggest the association of rs10735079 (risk allele G) with the severity of SARS-CoV-2 infection (odds ratio (OR) = 1.3) [12,21].

MIS-C and Kawasaki disease (KD) show similarities in their pathogenesis (autoimmune pattern), clinical course (fever, rash, no purulent conjunctivitis, cardiovascular involvement) and laboratory findings (elevated pro-inflammatory markers and evidence of coagulopathy) [22]. Therefore, in differential diagnosis, we assessed genome-wide significant variants associated with KD susceptibility [22]. Current studies suggest 63 genes associated with KD [23]. Among them, four groups of genes that are related to KD susceptibility were formed: (1) genes that enhance T cell activation (ITPKC, ORAI1, STIM1); (2) genes related to the dysregulation of B cell signaling (CD40, BLK, FCGR2A); (3) genes associated with decreased apoptosis (CASP3) and (4) genes related to altered transforming growth factor beta signaling (TGFB2, TGFBR2, MMP, SMAD) [23,24]. All listed genes have different risk allele frequencies in the European and Asian populations; therefore, we have focused on the genes that have a higher frequency in Europe—CD40 (CD40 molecule), Fc gamma receptor IIa (FCGR2A) and caspase 3 (CASP3) [22]. In addition, these genes are among the most important regulators of antiviral immune response [25]. CD40 as a costimulatory surface receptor is expressed on B-cells, macrophages, monocytes, platelets, dendritic cells and non-hematopoietic cells—vascular endothelial cells, epithelial cells, myofibroblasts and fibroblasts [26,27,28]. Therefore, CD40 contributes to cellular and humoral immunity as well as to vascular remodeling [26,27,29]. FCGR2A (Fc fragment of immunoglobulin G, low-affinity IIa, receptor) is also discussed in the context of immune response and vascular remodeling [30]. Polymorphism FCGR2A rs1801274 is associated with the substitution of histidine by arginine at position 131 (H131R) [30]. As a result, binding affinity with different immunoglobulins G subclasses is changed and the autoimmune response is activated [30]. Caspase-3 is a well-known protein involved in apoptosis and it is encoded by the CASP3 gene. The activation of caspase-3 in COVID-19 patients can be caused by the enhanced production of reactive oxygen species in the presence of oxidative stress. At the same time, the cytokine storm in SARS-CoV-2 infection is described as the result of apoptosis-related cellular death [31].

Therefore, CD40, FCGR2A and CASP3 genes are discussed as the genetic predisposition factors of KD, as well as severe COVID-19 and MIS-C, in children.

Despite the number of genetic studies related to COVID-19 severity and outcome, the results are controversial, with different findings and associations presented. However, no previous analyses regarding the roles of genetic factors in COVID-19 severity in children have been reported. Therefore, it is the focus of our research.

## 2. Materials and Methods

A total number of 75 children were involved in the case–control study—30 persons with mild or moderate course of the disease; 30 children with severe COVID-19 and multisystem inflammatory syndrome and 15 healthy children who did not have COVID-19 (PCR-negative, Ig G-negative).

Criteria of the Italian Society of Pediatric Infectious Disease [32,33] and COVID-19 Treatment Guidelines (National Institutes of Health) [34] were used to define disease severity. Mild disease severity was diagnosed in cases when upper airway symptoms without radiological/ultrasound findings were present despite the temperature level. Children with pneumonia diagnosed by imaging studies or persons with upper airway symptoms with respiratory distress were defined as patients with moderate disease severity. When patients had a fever or cough with saturation <92% in room air, severe respiratory distress or systemic symptoms (drowsiness, lethargy, seizures, dehydration), severe disease course was diagnosed. 

Diagnosis of MIS-C was done based on the World Health Organization (WHO) criteria [35]—fever duration greater than three days in children and adolescents (0–19 years), plus two of the following: (1) rash or bilateral nonpurulent conjunctivitis or mucocutaneous inflammation signs (oral, hands or feet); (2) hypotension or shock; (3) features of myocardial dysfunction, pericarditis, valvulitis or coronary abnormalities (including ECHO findings or elevated Troponin/N-Terminal Pro–B-Type Natriuretic Peptide [NT-proBNP]); (4) evidence of coagulopathy (by prothrombin time, partial thromboplastin time, elevated d-Dimers) or (5) acute gastrointestinal problems (diarrhea, vomiting or abdominal pain). Other evaluations used in MIS-C diagnosis included elevated markers of inflammation such as erythrocyte sedimentation rate, C-reactive protein or procalcitonin, and no other obvious microbial cause of inflammation, including bacterial sepsis, staphylococcal or streptococcal shock syndromes, as well as evidence of COVID-19 (real-time reverse transcription polymerase chain reaction, antigen test or serology positive) or likely contact with patients with COVID-19.

Children enrolled in the study were examined in Ternopil, Ukraine (Ternopil Municipal Children’s Hospital and Ternopil Regional Children’s Clinical Hospital).

The study was conducted and performed under the principles of the Declaration of Helsinki. The Bioethics Committee of I. Horbachevsky Ternopil National Medical University approved this study (Protocol No 71, dated 25 October 2022). Informed consent was obtained from all the children’s caregivers.

Venous blood samples for the genomic study were collected in tubes with ethylenediamine tetra acetic acid. The blood sample volumes used for clinical care and research did not exceed the recommended limit set by the WHO [36]. The Thermo Scientific™ GeneJET™ Whole Blood Genomic DNA Purification Mini Kit Cat. No K0781 (Thermo Fisher Scientific, Waltham, MA, USA, 02451) was used for genomic DNA extraction according to the manufacturer’s instructions. Predesigned TaqMan™ SNP Genotyping Assays, Cat. No. 4351379 (Thermo Fisher Scientific, Waltham, MA, USA, 02451), were used for the following SNPs—ACE2 rs2074192, IFNAR2 rs2236757, TYK2 rs2304256, OAS1 rs10774671, OAS3 rs10735079, CD40 rs4813003, FCGR2A rs1801274 and CASP3 rs113420705. A TaqMan™ Universal Master Mix II, no UNG, 1 × 5 mL, Cat. No 4440040 was used for DNA amplification using real-time polymerase chain reaction.

Statistical analysis was performed with the computer software IBM SPSS Statistics 21.0. Quantitative values are presented as number (*n*) and frequency (%). Frequency tables 2 × 3 were compared using the chi-square test (χ^2^), while for tables 2 × 2, the two-tailed Fisher exact test was used; the level of significance for each test was calculated as the *p*-value and p_F_, respectively. Correspondence for Hardy–Weinberg equilibrium was assessed for each gene. With the assumption that gene frequency corresponds to the population, a measurement was taken when *p* > 0.05 in the chi-square test. For outcome prediction, OR with its 95% confidence interval (95% CI) was calculated. Logistic regression was performed to determine the key predictors of COVID-19 and its severity in the pediatric population. A level of statistical significance was assumed with a *p*-value < 0.05. GeneMANIA network data were used to assess the network cooperation between studied genes [37]. The statistical tool “G*Power 3.1.9.7” was used for sample size calculations and post-hoc power analysis. For determining the total sample size, we used goodness-of-fit tests for contingency tables (χ^2^ tests). We assumed an effect size at the level of 0.5 (medium effect according to Cohen’s criteria). Statistical power was fixed at 0.8. The degree of freedom was chosen based on two different planned study approaches: (1) three groups (two COVID-19 groups and one control group)—Df = 2; (2) three groups (two COVID-19 groups and one control group) with different 3 genotypes—Df = 4. Based on the obtained results the required total sample size was fixed as 75 persons. The post-hoc power analysis revealed statistical power, which exceeded 0.8 for genes ACE2, IFNAR2, OAS1, OAS3, CD40, and CASP3, and confirmed that the precise number of participants used was the sufficient sample size. The input parameters for the power calculations were total sample size (75 persons), an α error of probability of 0.05, and an effect size index w that was calculated based on the obtained results (w ACE = 0.45; w IFNAR2 = 0.40; w TYK2 = 0.26; w OAS1 = 0.81; w OAS3 = 0.36; w CD40 = 0.35; w FCGR2A = 0.30; w CASP3 = 0.45). The study results demonstrate the statistical powers for eight selected genes—(1) ACE2 rs2074192 power = 0.97; (2) IFNAR2 rs2236757 power = 0.94; (3) TYK2 rs2304256 power = 0.62; (4) OAS1 rs10774671 power = 0.99; (5) OAS3 rs10735079 power = 0.36; (6) CD40 rs4813003 power = 0.86; (7) FCGR2A rs1801274 power = 0.75; and (8) CASP3 rs113420705 power = 0.97.

## 3. Results

### 3.1. Study Group’s Characteristic

The demographic characteristics of children involved in the study are presented in Table 1. There was no sex difference in the studied group, while an age difference was revealed. Children with mild or moderate COVID-19 were significantly younger compared to the control group. Age among COVID-19 patients did not vary (Table 1).

### 3.2. Correspondence to Hardy–Weinberg Equilibrium

The frequency of ACE2 rs2074192, IFNAR2 rs2236757, OAS3 rs10735079, FCGR2A rs1801274 and CASP3 rs113420705 genotypes did not follow the Hardy–Weinberg equilibrium (*p* < 0.05) due to the directional selection made in our study (focusing on patients with COVID-19). In the COVID-19 group, the TYK2 rs2304256, OAS1 rs10774671, CD40 rs4813003 and FCGR2A rs1801274 homozygote and heterozygote frequencies were in accordance with those indicated by the Hardy–Weinberg principle (*p* > 0.05). Notably, genotype frequencies in healthy children (control group) correspond to the Hardy–Weinberg proportions (*p* > 0.05) (Supplementary Materials: Tables S1–S3).

### 3.3. Genotype and Allele Frequencies

#### 3.3.1. ACE2 rs2074192

**Figure 1:**
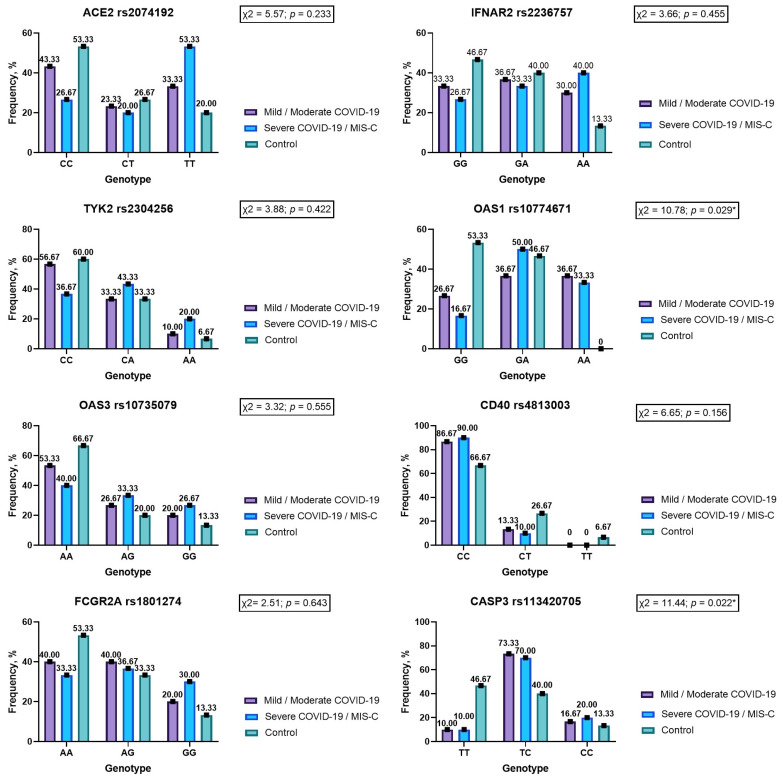
Genotype frequencies in the codominant model for genes ACE2 rs2074192, IFNAR2 rs2236757, TYK2 rs2304256, OAS1 rs10774671, OAS3 rs10735079, CD40 rs4813003, FCGR2A rs1801274 and CASP3 rs113420705 in children with COVID-19 and noninfected children (ACE 2—angiotensin converting enzyme 2; IFNAR2—interferon alpha and beta receptor subunit 2; TYK2—tyrosine kinase 2; OAS1—2′-5′-oligoadenylate synthetase 1; OAS3—2′-5′-oligoadenylate synthetase 3; CD40—CD40 molecule; FCGR2A—Fc gamma receptor IIa; CASP3—caspase 3; *—statistically significant result.).

The genotype frequencies did not vary in the studied group in the codominant model (Figure 1). Analyses of dominant, recessive and overdominant models demonstrate that children with severe COVID-19 or MIS-C are more often the carriers of the TT genotype ACE2 rs2074192. Genotype TT rs2074192 increases the risk of having a severe course of SARS-CoV-2 infection by 4.57 times (OR = 4.57; 95% CI 1.07–19.57; *p* = 0.041) (Table 2).

Children with severe COVID-19 course and MIS-C are significantly more often allele T carriers ACE2 rs2074192 compared to healthy persons (*p* = 0.017). Allele T increases the risk of severe disease course by 3.45 times (*p* = 0.009), while allele C has protective properties (OR = 0.29; 0.12–0.73) (Table 3).

The comparison of our results with data from the project “ALFA: Allele Frequency Aggregator” [38] shows that the frequency of alleles C and T in healthy children corresponds to the average frequency in the European population (*p* = 0.061), while patients with COVID-19 are more often carriers of risk allele T compared to the general population (0.54 versus 0.45; *p* = 0.048) (Table 4).

Notably, the genotype frequency varied significantly between girls and boys in the COVID-19 group—boys showed genotype TT more often compared to girls. In healthy children, this sex difference was not revealed (Table 5). At the same time, a comparison of healthy boys and boys with COVID-19 showed a significant difference in ACE2 rs2074192 genotype frequencies (χ^2^ = 6.94; *p* = 0.031); healthy children and those infected with SARS-CoV-2 girls did not differ in terms of genotype frequency (χ^2^ = 0.68; *p* = 0.711) (Table 5). Allele T frequency varied between groups—it was found in 60.26% of infected boys compared to 14.29% of noninfected boys (*p* = 0.002) (Table 5).

#### 3.3.2. IFNAR2 rs2236757

Rare homozygous AA was seen in 40% of patients with severe COVID-19 and MIS-C, while in the control group it was observed in 13.33% of cases. Despite the higher frequency, this difference did not reach the level of statistical significance (*p* = 0.071) (Figure 1). In the recessive inheritance model, genotype AA IFNAR2 rs2236757 showed a tendency to increase disease severity more than fourfold (OR = 4.33; 95% CI 0.83–22.75; *p* = 0.083) (Table 6).

IFNAR2 rs2236757 risk A allele was revealed significantly more often among patients with severe COVID-19 and MIS-C compared to the control group—56.67% vs. 33.33% (*p* = 0.046). Carriers of risk allele A are more prone to suffer from severe COVID-19 or MIS-C (OR = 2.62; *p* = 0.039) (Table 3).

It is important to emphasize that allele A is registered significantly more often in children with COVID-19 compared to data in the general European population (*p* < 0.001), while the frequencies of alleles G and A in healthy children correspond to it (*p* = 0.497) (Table 4).

There were no sex differences in genotype or allele frequencies between the COVID-19 group and control group (*p* > 0.05). However, comparison in terms of the same sex demonstrates a higher frequency of risk allele A in boys with COVID-19 compared to healthy boys—50% and 21.43% (*p* = 0.049); in girls, such a difference was not revealed (*p* = 0.365) (Table 5).

#### 3.3.3. TYK2 rs2304256

There were no differences in TYK2 rs2304256 genotype frequencies according to the codominant, dominant, recessive and overdominant models (*p* > 0.05) (Figure 1, Table 7).

The frequencies of alleles C and A also did not vary between groups with different COVID-19 severity and healthy children (Table 3). Risk allele A was seen in 23.33% of healthy persons vs. 41.67% in patients with severe COVID-19/MIS-C and 26.67% in the mild/moderate group (*p* > 0.05). Allele frequencies in the COVID-19 group were similar to those in the general population (Table 4).

The TYK2 rs2304256 genotype and allele frequencies were similar between boys and girls, as well as between infected and noninfected children of the same sex (*p* > 0.05) (Table 5).

#### 3.3.4. OAS1 rs10774671

The codominant model’s study revealed differences in genotype frequencies for gene OAS1 rs10774671 (*p* < 0.05) (Figure 1). Genotype AA OAS1 rs10774671 was registered in 35% of patients infected by SARS-CoV-2, while in healthy children it was not noticed (*p* = 0.022). Our study results demonstrate that homozygous GG is more than five times less likely to have a severe COVID-19 course (OR = 0.18) compared to carriers of allele A (heterozygous GA and homozygous AA)—OR = 5.71 (*p* < 0.05) (Table 8).

Patients with COVID-19 were often a carrier of allele A (56.67%) compared to healthy children (23.33%) (*p* = 0.001) (Table 3). Notably, allele A OAS1 is associated with severe COVID-19 course (OR = 4.60; 95% CI 1.71–12.37; *p* = 0.003). It should be noted that patients in the control group did not match the European population in terms of OAS1 rs10774671 allele G and A frequencies (Table 4).

Frequencies of genes located on the autosome OAS1 rs10774671 genotype and alleles were similar for both sexes (Table 5).

#### 3.3.5. OAS3 rs10735079

There were no statistical differences between OAS3 rs10735079 genotypes and allele frequencies between children with different degrees of COVID-19 severity and healthy children (Figure 1, Table 3 and Table 9). Nevertheless, it should be noticed that the frequencies of allele G and genotypes with allele G (GG + GA) were two times higher in persons with a severe COVID-19 course compared to the control group—43.33% vs. 23.33% for alleles (*p* = 0.065) and 60% vs. 33.33% for genotypes (*p* = 0.095).

OAS3 rs10735079 allele frequencies in noninfected children differ significantly from the results for the European population (*p* = 0.024) (Table 4). Sex differences were not typical for rs10735079 (Table 5). 

#### 3.3.6. CD40 rs4813003

The research did not reveal significant differences in codominant, dominant, recessive and overdominant models between study groups for gene CD40 rs4813003 (Figure 1, Table 10). Importantly, genotype CC was typical for 90% of children with severe COVID-19 and MIS-C, while in a healthy group it was registered in 66.67% of cases (*p* = 0.056). Correspondingly, allele C significantly dominated in the COVID-19 group compared to noninfected children—94.17% vs. 80% (*p* = 0.024). Based on our study results, we can infer that allele C increases the risk of severe COVID-19 course or MIS-C 4.75 times (*p* = 0.037) (Table 3).

It is important to note that allele C and T frequencies were significantly different here compared not only to the control but also to the general European population (*p* = 0.011). In noninfected children, the frequencies of both allele C and T correspond to those seen in the general population (*p* > 0.05) (Table 4).

The comparison between boys and girls did not show any difference in genotype or allele frequency (*p* > 0.05) (Table 5).

#### 3.3.7. FCGR2A rs1801274

The analysis of all genetic models of inheritance did not reveal significant changes in FCGR2A rs1801274 genotype frequencies between those infected by the SARS-CoV-2 virus and noninfected persons (Figure 1, Table 11).

Allele A and G frequencies in patients with COVID-19 did not differ significantly from the control group or from the general population (*p* > 0.05) (Table 3 and Table 4). At the same time, our study results demonstrate a deviation in allele frequencies between healthy children and the European population (*p* < 0.05) (Table 4).

Genotype and allele frequencies in the male group correspond to the same parameters in the female group of COVID-19 patients and healthy children (*p* > 0.05) (Table 5).

#### 3.3.8. CASP3 rs113420705

CASP3 rs113420705 genotype TT was registered significantly more often among healthy children (46.67%) compared to patients with COVID-19 of levels of different severity (10%) (*p* < 0.05) (Figure 1). Notably, TT genotype rs113420705 is protective against severe COVID-19 or MIS-C in the pediatric population (OR = 0.13; *p* = 0.010) (Table 12). Carriers of allele C in both heterozygous TC and rare homozygous CC have an increased risk of severe SARS-CoV-2 infection (OR = 7.88) (Table 12). Important to note is that despite the absence of a statistically significant difference between COVID-19 groups and the control, the study noted a significantly higher prevalence of allele C in COVID-19 pediatric patients and European allele frequencies (Table 3 and Table 4).

Sex differences were not typical for CASP3 rs113420705 genotypes and allele frequencies (Table 5).

#### 3.3.9. Allele Associations and Gene Interrelationships in Children with COVID-19

Our research has revealed that children not infected by SARS-CoV-2 are often the carriers of two or three risk alleles (*p* < 0.001). However, 30% of COVID-19 patients are carriers of seven to eight risk alleles, which is not typical for healthy children (*p* = 0.034) (Table 13).

The logistic regression model for COVID-19 prediction based on the child’s sex and alleles was statistically significant (χ^2^ = 45.96; *p* < 0.001 *) (Table 14). The proposed model explained 41.7% (Nagelkerker R Square) of the variation in COVID-19 susceptibility and correctly predicted 85.3% of cases. Therefore, based on the model, the key components of COVID-19 susceptibility in childhood are alleles of genes IFNAR2 rs2236757, OAS1 rs10774671, OAS3 rs10735079, CD40 rs4813003 and CASP3 rs113420705.

Such indicators as male sex, ACE2 rs2074192 allele T, IFNAR2 rs2236757 allele A, OAS1 rs10774671 allele A, CD40 rs4813003 allele C and CASP3 rs113420705 allele C can predict severe COVID-19 course and MIS-C in the pediatric population in 85.6% of cases (Table 15). The proposed model is significant (χ^2^ = 56.85; *p* < 0.001) and demonstrates strong goodness of fit—Nagelkerke R square 0.65.

**Figure 2:**
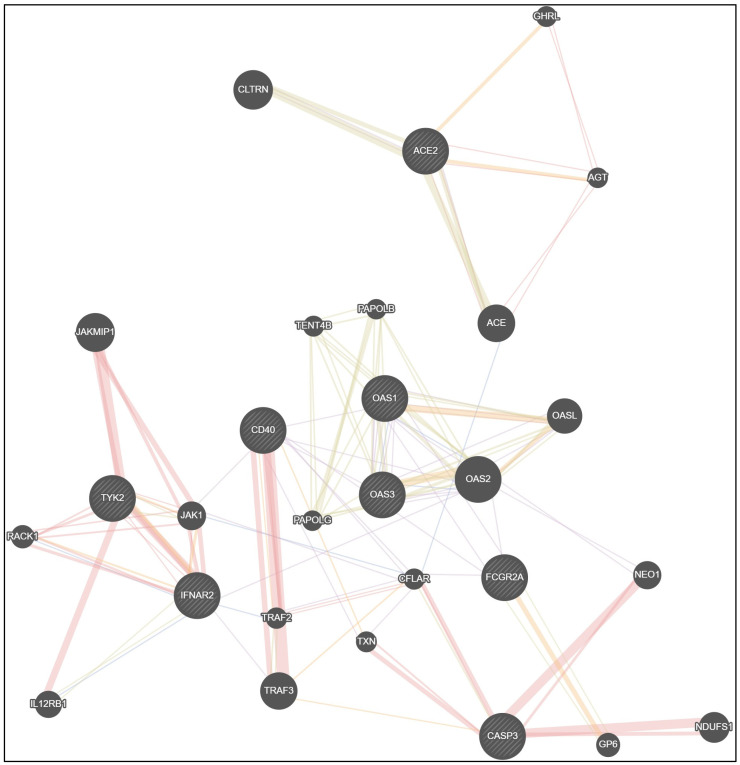
Network data for gene interactions between studied genes ACE2, IFNAR2, TYK2, OAS1, OAS3, CD40, CASP3, FCGR2A and other genes generated by GeneMANIA.

Network data from GeneMANIA [37] confirm the interrelationship between the studied genes (Figure 2). It should be emphasized that in immune protection, many genes are involved. With the exception of eight studied genes (ACE2, IFNAR2, TYK2, OAS1, OAS3, CD40, CASP3, FCGR2A), we must also focus on the genes angiotensin I converting enzyme (ACE), angiotensinogen (AGT), collectrin, amino acid transport regulator (CLTRN), ghrelin and obestatin prepropeptide (GHRL), 2′-5′-oligoadenylate synthetase 2 (OAS2), 2′-5′-oligoadenylate synthetase like (OASL), poly(A) polymerase beta (PAPOLB), poly(A) polymerase gamma (PAPOLG), terminal nucleotidyltransferase 4B (TENT4B), TNF receptor associated factor 2 (TRAF2), TNF receptor associated factor 3 (TRAF3), CASP8 and FADD like apoptosis regulator (CFLAR), thioredoxin (TXN), neogenin 1 (NEO1), glycoprotein VI platelet (GP6), NADH: ubiquinone oxidoreductase core subunit S1 (NDUFS1), Janus kinase 1 (JAK1), Janus kinase and microtubule interacting protein 1 (JAKM1P1), receptor for activated C kinase 1 (RACK1) and interleukin 12 receptor subunit beta 1 (IL12RB1) (Figure 2).

Physical interactions between genes in the proposed network model were registered in 35.92% of cases, co-expression in 30.46%, shared protein domains in 17.70%, co-localization in 4.55% and predicted functional relationships between genes in 11.37% of cases (Table 16).

## 4. Discussion

The research conducted suggests the importance of host genetic factors in antiviral immunity against SARS-CoV-2 in children. It is clear that genetic patterns are stable during the whole of life; despite this, the influence of SNPs on COVID-19 severity was studied first. We focused on the key genes, which play the most significant role in the immune response in COVID-19, and we studied genes related to Kawasaki disease.

Our study results confirm certain genes’ interrelationships and how they predict the course of SARS-CoV-2 infection. Regression analysis showed that COVID-19 susceptibility in children increases in cases of interactions of IFNAR2 rs2236757, OAS1 rs10774671, OAS3 rs10735079, CD40 rs4813003 and CASP3 rs113420705. Crucial factors for the development of severe COVID-19 or MIS-C are sex (male gender), ACE2 rs2074192, IFNAR2 rs2236757, OAS1 rs10774671, CD40 rs4813003 and CASP3 rs113420705.

More often, pediatric patients with COVID-19 were found to be the carriers of a combination of seven or eight risk alleles. Noninfected children were more likely to be the carriers of three or fewer risk alleles compared to patients with COVID-19 of different severity.

Importantly, our study has demonstrated the significant prevalence of risk alleles among pediatric COVID-19 groups compared to relevant data for the European population. These frequency differences were revealed for four of the studied genes—ACE2 rs2074192 allele T (54% vs. 45%), IFNAR2 rs2236757 allele A (52% vs. 29%), CD40 rs4813003 allele C (94% vs. 86%) and CASP3 rs113420705 allele C (54% vs. 28%). This finding is of great importance in the confirmation of genetic susceptibility to SARS-CoV-2 infection.

Gene ACE2 encodes cognominal type 1 membrane protein ACE2 [8]. The ACE2 gene is located on the X chromosome (Xp22.2), locus NG_012575 [39,40]. Numerous studies suggest that single-nucleotide polymorphisms (SNPs) in the gene ACE2 are related to the expression of ACE2 and the following binding affinity of SARS-CoV-2 [41,42].

Our research revealed sex differences in risk allele T frequency—boys are more often the carriers of it; therefore, they are more susceptible to SARS-CoV-2 infection. Such sex differences can be explained by the location of ACE2 rs2074192 on the X chromosome. The study suggests that such Xp22 gene location encompasses an area that is not under physiological X-inactivation [8]. X-inactivation normally happens in females to ensure population basis gene distributions. Escaping from X-inactivation leads to different phenotypic patterns and sex tissue-specific differences [8].

Allele T of ACE2 rs2074192 shows a higher frequency in symptomatic COVID-19 patients compared with the asymptomatic group, and it is more often associated with severe outcomes [43]. Previous meta-analyses performed by K. Gupta et al. demonstrated genotype contrasts between the TT and CT genotypes ACE2 rs2074192 in severe COVID-19 prediction in an adult population, but no allele differences [44]. Our research received similar results. However, our study also demonstrated the influence of allele differences and risk allele T on the SARS-CoV-2 infection course in children. In L. E. Martinez-Gomez et al.’s study, codominant, dominant and recessive models did not reveal significant differences between severe and critical COVID-19 patients [45]. Research in Spain demonstrated the protective effect of ACE2 rs2074192 in relation to female hospitalization during COVID-19 [40].

Therefore, data regarding ACE2 rs2074192’s impact on SARS-CoV-2 are still controversial, and future studies must be carried out.

The SARS-CoV-2 virus has a direct stimulatory effect on interferon-stimulated genes (ISG), and also causes the activation of immune cells. ISGs cause an increase in the expression of pro-inflammatory genes. Type I interferon response is among the key pathogenetic bases for antiviral host defense. The course of COVID-19 is characterized by the reduced production of IFN-I in the early stages of the disease. At the same time, studies have emphasized the role of the interferon system in inducing a “cytokine storm” by activating the synthesis of TNF/IL-1β [46]. Hadjadj et al. demonstrated that patients with severe COVID-19 display a downregulation of IFN-stimulated genes (MX1, IFITM1 and IFIT2) compared to patients with mild and moderate disease severity [47,48,49]. Research has suggested that a low type I IFN response manifests a worsening of the clinical condition up to the critical state [17,47]. At the same time, endogenous or exogenous steroids can suppress IFN signaling that manifest impaired antiviral immune responses and increasing disease severity. This is crucial to our understanding of the COVID-19 course, because glucocorticoids are used in the medical management of MIS-C, acute respiratory distress syndrome and sepsis [50]. Therefore, the INF pathway and its genetic control should be assessed in the pediatric population.

Intron variant IFNAR2 rs2236757 is located on chromosome 21q22.1 (chr21:34,624,917) [12]. Fricke-Galindo et al. showed the association of IFNAR2 rs2236757 with disease severity and mortality risk in adults with COVID-19 [13]. The study of genetic mechanisms critical to COVID-19 also underlined that the low expression of IFNAR2 is associated with life-threatening disease, while its high expression reduces the odds of severe COVID-19 [12]. The association of the AA genotype rs2236757 with intensive care admission was shown in adult patients infected by SARS-CoV-2 [16,17].

Despite the fact that mortality rate and intensive care unit admission were not the focus of our research, our findings in a pediatric population are in line with public data regarding disease severity—allele A increases the risk of severe COVID-19 and MIS-C in single-gene studies and in cases of multigene interaction.

Tyrosine kinase 2 (TYK2) is a protein functioning as a Janus kinase/signal transducer and an activator of transcription (Jak-STAT) pathways [14]. TYK2 activation is carried out by IFN-α, which leads to the phosphorylation of STAT1 and STAT2 and the subsequent dimerization of activated STATs. The subsequent translocation of dimerized STATs in the cell nucleus leads to the induction of interferon-stimulated gene expression and the activation of antiviral defense [15,51]. TYK2 also stimulates nontraditional pathways of antiviral protection, such as NF-κB signaling and the mitogen-activated protein kinase pathway [51]. TYK2 regulates the activity and function of T-helpers of type 1 and T-helpers of type 17 through a functional connection with IL-12 and IL-23 [15,52]. The differentiation of CD4+ T cells, activated by the interaction of IL-23 with TYK2/Jak2, occurs with the participation of IL-6, IL-1β and TGF-β, which is extremely important in the context of the cytokine storm in COVID-19 [52].

TYK2 (rs2304256) is a gene that encodes a non-receptor tyrosine-protein kinase; it is a nonsynonymous variant that is located on chromosome 19 in exon 8 (chr19:10,350,533–10,380,608) [53,54]. It is suggested that the TYK2 gene is associated with susceptibility to inflammatory and autoimmune disorders [53]. In the European population, the protective role of minor allele A was demonstrated in systemic lupus erythematosus, type 1 diabetes, psoriasis and idiopathic inflammatory myopathies [53]. Associations with autoimmune diseases can be explained by the rs2304256-related modification of expression of less common disease variants rs34536443 (P1104A) and rs12720356 (I684S) [53].

Our research did not find any differences in genotype or allele frequencies in TYK2 rs2304256 depending on COVID-19 severity. This can be explained by the previously reported absence of altered TYK2 function after acid substitution caused by rs2304256 [53]. Therefore, based on our study, TYK2 did not impact COVID-19 outcome or the autoimmune regulatory mechanism that can be defined in MIS-C, but studies are ongoing, and other regulatory pathways could be found.

The OAS gene family is closely related with interferon-stimulated genes (ISG) [46]. OAS genes are located on chromosome 12 (12q24.13 region) and encode the synthesis of oligoadenylate OAS1, OAS2 and OAS3 [19].

Activated by viral RNA (mainly double-stranded), oligoadenylate catalyzes ATP polymerization and the activation of latent ribonuclease (RNase L) [55]. Direct Rnase L action leads to viral RNA destruction [19,56]. OAS–Rnase L cleaves viral messenger RNA, and as a result, viral replication is impossible [55]. RNase L activation leads to ribosomal and mitochondrial RNA degradation, and then apoptosis [57].

The most recent studies suggest that single-gene inborn errors of the OAS-RNase L lead to the uncontrolled production of pro-inflammatory cytokines by mononuclear phagocytes, which can predispose one to MIS-C development [58]. Lee et al. revealed that approximately 1% of patient with MIS-C had autosomal recessive deficiencies of OAS1, OAS2 or RNase L [58].

The results of our study demonstrate the protective effect of GG genotype OAS1 (rs10774671) in a dominant inheritance model of severe COVID-19 (OR = 0.18; *p* < 0.05), while carriers of allele A showed a higher risk of worse COVID-19 outcome (OR = 5.71; *p* < 0.05). The influences of allele A OAS1 rs10774671 on COVID-19 susceptibility and its severe course were confirmed by two logistic models. Our data correspond to the previously presented data from an adult population, where the involvement of rs10774671 in SARS-Co-V-2 pathogenesis was demonstrated [55]. The presence of allele A is associated with two mRNA variants—p48 and p52, with low OAS activity. Allele G leads to the production of the p46 form with high OAS activity [55].

Studies show that the presence of the risk allele G rs10735079 gene leads to reductions in the OAS1 level, which proves the existence of a negative inverse relationship between the severity of the course of COVID-19 and the level of OAS1 [21]. The level of OAS1 is directly related to hospitalization frequency in cases of diagnosed pneumonia [5,21]. However, the role of oligoadenylate synthases in the elimination of single-stranded viral RNAs remains debatable, and requires further study. Before now, results regarding OAS3 rs10735079 have been controversial. Horowitz et al. performed comparisons between SARS-CoV-2-positive patients vs. SARS-CoV-2-negative or unknown, and reported that allele G was the allele affecting COVID-19 pathogenesis [59]. At the same time, Pairo-Castineira et al. as well as Pietro et al. presented the influence of allele A rs10735079 on COVID-19 manifestations in adults and in children [5,12].

Our study did not reveal any differences in the genetic models between the studied OAS3 rs10735079 groups. However, in the case of gene interactions, the significant impact of OAS3 rs10735079 was shown on SARS-CoV-2 susceptibility, but it was not shown to be involved in the prediction of MIS-C or severe COVID-19 development. Importantly, the frequency of minor allele G in our research was significantly lower in healthy children compared to the European population. This diversity can be explained by the small sample in our control group (*n* = 15). Therefore, the following comparisons between allele frequencies in infected and noninfected groups were difficult.

CD40 is a 48-kDa type I transmembrane protein and belongs to the tumor necrosis factor (TNF) receptor (TNFR) family [26]. It was revealed that cytokine production is stimulated by CD40 engagement on the surface of dendritic cells [26]. Cell immunity is also mediated through CD40 signaling, and as a result, T-cell activation and differentiation are achieved [26]. At the same time, CD40 is involved in the regulation of the humoral immune response. B-cell activation by CD40 stimulates immunoglobulin (Ig) isotype switching and Ig somatic hypermutation [26]. As a result, the affinity of Ig to antigen is increased. The CD40-CD40L pathway is known as the regulator of the production of IL-10 and IL-12 by monocytes and macrophages [60]. Notwithstanding this, CD40-CD40L signaling is related to the formation of memory B cells, as well as long-lived plasma cells and their survival [26,61]. The dysregulation of CD40 is associated with the expansion of autoreactive B cells instead of their elimination, and leads to impaired immune response and autoimmune processes [60].

The CD40 molecule is encoded by the intergenic variant rs4813003 and is located on chromosome 20 (20:46134645; cytogenetic region 20q13.12) [62]. Current research suggests the rs4813003 TT genotype reduces the risk of KD (OR = 0.64), while genotypes CC and CT increase it [28]. Studies confirmed that the association of CD40 with KD is mainly related to the East Asian population, wherein risk allele C increases the risk of KD by 1.41 times [23,27,63]. In our research, CD40 rs4813003 genotype frequencies did not differ between study groups and the control, while allele C was seen significantly more often among patients with COVID-19 compared with the control (*p* < 0.05). Our study results also demonstrate the significant impact of allele C on severe COVID-19 course or MIS-C in both single-allele analyses (OR = 4.75; *p* = 0.037) and in case of allele interactions (OR = 264.57; *p* = 0.004).

Risk allele C increases not only CD40 function, but also increases the expression of its ligand CD40L [23,26]. Soluble CD40L is associated with vasculitis and vascular remodeling [64]. Most of the soluble sCD40L is of platelet origin, which indicates the activation of platelets in the lungs’ microcirculation during SARS-CoV-2 infection. sCD40L is able to locally activate endothelial cells, pericytes and smooth muscle cells, stimulate the expression of FGF-2, and, as a result, induce vascular wall remodeling [64]. In this regard, studies of CD40 rs4813003 polymorphism are important in relation to COVID-19 and MIS-C because cardiovascular system involvement is typical for both of them, especially MIS-C.

Missense variant rs1801274 encodes transmembrane glycoprotein Fc gamma (γ) receptor IIa and is located on chromosome 1 (1:161505430–161524013, cytogenetic region 1q23.3) in the EC2 domain [62,65,66].

Fc γ receptor (FcγR) presents on the immune cells and can bind with specific antibodies. Complex FcγR and Ig G stimulate the release of cytokines, the production of reactive oxygen species, as well as antibody-dependent cellular cytotoxicity and phagocytosis [65,66]. Based on its affinity to different Ig G subtypes, FcγR is divided into three types: FCGR1, FCGR2 and FCGR3. FCGR2 has the lowest affinity to Ig G. Three different subtypes of FCGR2 were revealed—A, B and C. FCGR2 is presented on a variety of immune competent cells, such as natural killers, macrophages and neutrophils [65]. Variable responses to infection depend on Ig F’s binding affinity. Polymorphism in the rs1801274 variant leads to the formation of two different isoforms of FCGR2—FcγRIIA-Arg and FcγRIIA-His [66]. These two forms can be made via the substitution of histidine for arginine at position 131 of the FCGR2A protein [67]. FcγRIIA-His has a higher affinity to Ig G1 and Ig G2 compared to FcγRIIA-Arg, while the binding capacities of Ig G3 and Ig G4 are almost the same in both isoforms [66]. Therefore, any deviations in FCGR2A expression can lead to the implicated Ig G binding affinity and, consequently, to immune dysregulation. It is suggested that FCGR2A is responsible for the modulation of severity of infection and autoimmune response—it is not only a marker of KD [66]. It has also been proposed to use genetic studies of FCGR in disease management, especially in the case of the prescription of therapeutic antibodies [66].

Study results have shown that risk allele G of gene FCGR2A rs1801274 increases the risk of KD under both the homozygous (AA) and heterozygous (AG) models in Asian and Caucasian populations, while allele A has a protective effect [65,68]. At the same time, controversial results were demonstrated by Zhang et al., whereby the estimation of the association between the FCGR2A rs1801274 polymorphism in Asian and Caucasian populations revealed that risk allele A is related to KD (OR = 1.41; *p* < 0.001) [69]. Research on adults of European ancestry demonstrated that allele G carriers (FcγRIIA-Arg) are more prone to lethal outcome of COVID-19 compared to homozygous AA carriers (OR = 2.2; *p* = 0.01) [66]. Greek researchers (Chatzikyriakidou et al.) did not reveal any genotype or allele differences between groups that suffered from KD and a healthy control [30]. Cardiovascular events were also not related to rs1801274 polymorphism [30]. Analyses of the previous thirteen case–control studies did not reveal a significant association between FCGR2A rs1801274 and overall pneumonia risk [67].

Our results correspond to those of European studies. The absence of a difference between the control group and the COVID-19 group, as well as the absence of a difference between COVID-19 patients and European FCGR2A allele frequencies, demonstrate that this gene is not related to disease severity.

Caspase 3 (CASP3) is a protein in the cysteine-aspartic acid protease (Caspase) family [66]. CASP3 with caspase-2, -6, -7, -8 and -9 is an apoptotic caspase [70]. An increase in CASP3 level is associated with increased apoptosis [70,71]. CASP3 could restrain IFN production. CASP3 deficiency is related to enhanced type I IFN secretion and the stimulation of innate immune response, and as a result, makes cells virally resistant [70]. CASP3 is also responsible for cell growth and differentiation, as well as for cytokine expression [68,72].

CASP3 is the gene located on chromosome 4 (4:184627696–184650062; cytogenetic region 4q35.1) that encodes protein caspase 3 [62]. Alterations in CASP3 expression lead to increased susceptibility to KD in European American and Asian populations [68]. A case–control study in North India demonstrated that persons with the CT genotype rs113420705 of CASP3 are more prone to have KD, and carriers of minor allele C most often have coronary artery aneurysm compared with T allele carriers [73]. Our study showed a significantly higher frequency of allele C rs113420705 in children with COVID-19 compared to those of European ancestry.

CASP3 gene is also important for the prevention of injuries caused by viral infection (SARS-CoV-2, hepatitis C virus, Enterovirus 71 infection, H-1 parvovirus) because CASP3 maintains cellular homeostasis and viability [72]. Therefore, it is suggested that the caspase’s level and its gene expression could be used to predict viral disease severity. In addition, hematological and immunological parameters of COVID-19 can be associated with dysregulated caspase activation [69]. In COVID-19 patients, CASP3 activity is upregulated in red blood cells, and platelet cell death can also be related to CASP3 [71].

The results of our study show the significant impact of CASP3 rs113420705 on SARS-CoV-2 susceptibility, and lead to a worse COVID-19 outcome in children.

## 5. Conclusions

COVID-19 shows different severity and outcomes in children. The clinical course varies from asymptomatic to mild and severe COVID-19 symptoms, and can even lead to MIS-C. Among the influencing factors, genetics is one of the most important. The carriers of pathogenic risk alleles are more prone to suffer from severe COVID-19. Allele T ACE2 rs2074192 (OR = 3.45; *p* = 0.009), allele A IFNAR2 rs2236757 (OR = 2.62; *p* = 0.039), allele A OAS1 rs10774671 (OR = 4.60; *p* = 0.003) and allele C CD40 rs4813003 (OR = 4.75; *p* = 0.037) are associated with severe COVID-19 that could advance to MIS-C. The multifactorial analysis shows that, in addition to ACE2, IFNAR2, OAS1, and CD40 genes, the CASP3 rs113420705 gene (allele C) makes the clinical outcome of COVID-19 in males worse. The findings of pathogenic risk alleles can help in the management of SARS-CoV-2 infections in children, and enhance COVID-19 prevention.

## Figures and Tables

**Table 1:** Demographic characteristics of children with COVID-19 (*n* = 60) and healthy children (*n* = 15).

Characteristic		Mild/Moderate COVID-19 (1)	Severe COVID-19/MIS-C (2)	Control (3)	*p*
Age, years	Median (Q25; Q75)	2.5 (1.0; 7.0)	7.0 (2.0; 14.0)	8.0 (5.0; 13.0)	p_1–2_ = 0.059 p_1–3_ = 0.011 * p_2–3_ = 0.943
	Min/Max	0.2/17.0	0.1/17.6	2.0/16.0	—
Sex	Boys	19 (63.33)	20 (66.67)	7 (46.67)	χ^2^ = 1.77; *p* = 0.413
	Girls	11 (36.67)	10 (33.33)	8 (53.33)	

Abbreviations: p_1–2_—statistical difference between group 1 (mild/moderate COVID-19) and group 2 (severe COVID-19/MIS-C); p_1–3_—statistical difference between group 1 (mild/moderate COVID-19) and group 3 (control); p_2–3_—statistical difference between group 2 (severe COVID-19/MIS-C) and group 3 (control); χ^2^—chi-squared test, *p*—level of its significance. *—statistically significant result.

**Table 2:** Dominant, recessive and overdominant models for ACE2 rs2074192.

Model	Genotype	Mild/ Moderate COVID-19	Severe COVID-19/MIS-C	Control	χ^2^, *p*-Value	OR (95% CI)	*p*-Value for OR
Dominant	CC	13 (43.33)	8 (26.67)	8 (53.33)	χ^2^ = 3.46 *p* = 0.177	0.32 (0.09–1.16)	0.084
	CT−TT	17 (56.67)	22 (73.33)	7 (46.67)		3.14 (0.86–11.50)	
Recessive	CC−CT	21 (70.00)	14 (46.67)	12 (80.00)	χ^2^ = 5.28 *p* = 0.071	0.22 (0.05–0.93)	0.041 *
	TT	10 (33.33)	16 (53.33)	3 (20.00)		4.57 (1.07–19.57)	
Overdominant	CC−TT	23 (76.67)	24 (80.00)	11 (73.33)	χ^2^ = 0.27 *p* = 0.875	1.45 (0.34–6.22)	0.613
	CT	7 (23.33)	6 (20.00)	4 (24.67)		0.69 (0.16–2.94)	

Abbreviations: χ^2^—chi-squared test; *p*-value—level of significance; OR—odds ratio (calculated for the outcome “severe COVID-19/MIS-C”); 95% CI—95% confidence interval (severe COVID-19/MIS-C—outcome in OR calculation). *—statistically significant result.

**Table 3:** Allele frequencies for genes ACE2 rs2074192, IFNAR2 rs2236757, TYK2 rs2304256, OAS1 rs10774671, OAS3 rs10735079, CD40 rs4813003, FCGR2A rs1801274 and CASP3 rs113420705 in children with different degrees of COVID-19 severity and noninfected children.

Gene	Allele	Mild/ Moderate COVID-19	Severe COVID-19/MIS-C	Control	χ^2^, *p*-Value	OR (95% CI)	*p*-Value for OR
ACE2 rs2074192	C	33 (55.00)	22 (36.67)	20 (66.67)	χ^2^ = 8.20; *p* = 0.017 *	0.29 (0.12–0.73)	0.009 *
	T	27 (45.00)	38 (63.33)	10 (33.33)		3.45 (1.37–8.69)	
IFNAR2 rs2236757	G	31 (51.67)	26 (43.33)	20 (66.67)	χ^2^ = 4.36; *p* = 0.113	0.38 (0.15–0.95)	0.039 *
	A	29 (48.33)	34 (56.67)	10 (33.33)		2.62 (1.05–6.53)	
TYK2 rs2304256	C	44 (73.33)	35 (58.33)	23 (76.67)	χ^2^ = 4.40; *p* = 0.111	0.43 (0.16–1.15)	0.091
	A	16 (26.67)	25 (41.67)	7 (23.33)		2.35 (0.87–6.31)	
OAS1 rs10774671	G	27 (45.00)	25 (41.67)	23 (76.67)	χ^2^ = 10.80; *p* = 0.005 *	0.22 (0.08–0.58)	0.003 *
	A	33 (55.00)	35 (58.33)	7 (23.33)		4.60 (1.71–12.37)	
OAS3 rs10735079	A	40 (66.67)	34 (56.67)	23 (76.67)	χ^2^ = 3.67; *p* = 0.159	0.40 (0.15–1.07)	0.068
	G	20 (33.33)	26 (43.33)	7 (23.33)		2.51 (0.94–6.75)	
CD40 rs4813003	C	56 (93.33)	57 (95.00)	24 (80.00)	χ^2^ = 6.19; *p* = 0.045 *	4.75 (1.10–20.57)	0.037 *
	T	4 (6.67)	3 (5.00)	6 (20.00)		0.21 (0.05–0.91)	
FCGR2A rs1801274	A	36 (60.00)	31 (51.67)	21 (70.00)	χ^2^ = 2.85; *p* = 0.241	0.46 (0.19–1.16)	0.100
	G	24 (40.00)	29 (48.33)	9 (30.00)		2.18 (0.86–5.54)	
CASP3 rs113420705	T	28 (46.67)	27 (45.00)	20 (66.67)	χ^2^ = 4.20; *p* = 0.122	0.41 (0.165–1.02)	0.056
	C	32 (53.33)	33 (55.00)	10 (33.33)		2.44 (0.98–6.10)	

Abbreviations: χ^2^—chi-squared test; *p*-value—level of significance; OR—odds ratio (calculated for the outcome “severe COVID-19/MIS-C”); 95% CI—95% confidence interval (severe COVID-19/MIS-C—outcome in OR calculation). *—statistically significant result.

**Table 4:** Comparison of allele frequencies in children infected by SARS-CoV-2 compared to the general European population.

Gene	Allele	Allele Frequency			p_COVID-19-EUR_	p_Control-EUR_
		Children with COVID-19	Healthy Children	European Population		
ACE2 rs2074192	C	0.46	0.67	0.55	0.048 *	0.061
	T	0.54	0.33	0.45		
IFNAR2 rs2236757	G	0.48	0.67	0.71	<0.001 *	0.497
	A	0.52	0.33	0.29		
TYK2 rs2304256	C	0.66	0.77	0.72	0.144	0.390
	A	0.34	0.23	0.28		
OAS1 rs10774671	G	0.43	0.77	0.36	0.110	<0.001 *
	A	0.57	0.23	0.64		
OAS3 rs10735079	A	0.62	0.77	0.63	0.818	0.024 *
	G	0.38	0.23	0.37		
CD40 rs4813003	C	0.94	0.80	0.86	0.011 *	0.342
	T	0.06	0.20	0.14		
FCGR2A rs1801274	A	0.56	0.70	0.51	0.271	0.039 *
	G	0.44	0.30	0.49		
CASP3 rs113420705	T	0.46	0.67	0.72	<0.001 *	0.542
	C	0.54	0.33	0.28		

Abbreviations: p_COVID-19-EUR_—level of statistical significance for comparison of COVID-19 group with general European population; p_Control-EUR_—level of statistical significance for the comparison of the control group (healthy children) with the general European population. *—statistically significant result.

**Table 5:** Sex differences in genotype frequencies among those infected with SARS-CoV-2 and noninfected children.

Gene	Genotype/Allele	COVID-19 (*n* = 60)		χ^2^, *p*/ p_F_	Control (*n* = 15)		χ^2^, *p*/ p_F_
		Boys	Girls		Boys	Girls	
ACE2 rs2074192	CC	13 (39.13)	8 (38.10)	χ^2^ = 6.95; *p* = 0.031 *	5 (71.43)	3 (37.50)	χ^2^ = 3.45; *p* = 0.178
	CT	5 (12.82)	8 (38.10)		2 (28.57)	2 (25.00)	
	TT	21 (53.85)	5 (23.81)		0	3 (37.50)	
	C	31 (39.74)	24 (57.14)	p_F_ = 0.085	12 (85.71)	8 (50.00)	p_F_ = 0.058
	T	47 (60.26)	18 (42.86)		2 (14.29)	8 (50.00)	
IFNAR2 rs2236757	GG	12 (30.77)	6 (28.57)	χ^2^ = 0.97; *p* = 0.615	4 (57.14)	3 (37.50)	χ^2^ = 2.09; *p* = 0.352
	GA	15 (38.46)	6 (28.57)		3 (42.86)	3 (37.50)	
	AA	12 (30.77)	9 (42.86)		0	2 (25.00)	
	G	39 (50.00)	18 (42.86)	p_F_ = 0.566	11 (78.57)	9 (56.25)	p_F_ = 0.260
	A	39 (50.00)	24 (57.14)		3 (21.43)	7 (43.75)	
TYK2 rs2304256	CC	20 (51.28)	8 (38.10)	χ^2^ = 1.25; *p* = 0.536	5 (71.43)	4 (50.00)	χ^2^ = 1.25; *p* = 0.535
	CA	13 (33.33)	10 (47.62)		2 (28.57)	3 (37.50)	
	AA	6 (15.38)	3 (14.29)		0	1 (12.50)	
	C	53 (67.95)	26 (61.90)	p_F_ = 0.548	12 (85.71)	11 (68.75)	p_F_ = 0.399
	A	25 (32.05)	16 (38.10)		2 (14.29)	5 (31.25)	
OAS1 rs10774671	GG	8 (20.51)	5 (23.81)	χ^2^ = 0.10; *p* = 0.953	4 (57.14)	4 (50.00)	p_F_ = 1.000
	GA	17 (43.59)	9 (42.86)		3 (42.86)	4 (50.00)	
	AA	14 (35.90)	7 (33.33)		0	0	
	G	33 (42.31)	19 (45.24)	p_F_ = 0.847	11 (78.57)	12 (75.00)	p_F_ = 1.000
	A	45 (57.69)	23 (54.76)		3 (21.43)	4 (25.00)	
OAS3 rs10735079	AA	19 (48.72)	9 (42.86)	χ^2^ = 1.06; *p* = 0.588	4 (57.14)	6 (75.00)	χ^2^ = 0.67; *p* = 0.715
	AG	10 (25.64)	8 (38.10)		2 (28.57)	1 (12.50)	
	GG	10 (25.64)	4 (19.05)		1 (14.29)	1 (12.50)	
	A	48 (61.54)	26 (61.90)	p_F_ = 1.000	10 (71.43)	13 (81.25)	p_F_ = 0.675
	G	30 (38.46)	16 (38.10)		4 (28.57)	3 (18.75)	
CD40 rs4813003	CC	34 (87.18)	19 (90.48)	p_F_ = 1.000	5 (71.43)	5 (62.50)	χ^2^ = 1.94; *p* = 0.379
	CT	5 (12.82)	2 (9.52)		1 (14.29)	3 (37.50)	
	TT	0	0		1 (14.29)	0	
	C	73 (93.59)	40 (95.24)	p_F_ = 1.000	11 (78.57)	13 (81.25)	p_F_ = 1.000
	T	5 (6.41)	2 (4.76)		3 (21.43)	3 (18.75)	
FCGR2A rs1801274	AA	12 (30.77)	10 (47.62)	χ^2^ = 3.00; *p* = 0.223	5 (71.43)	3 (37.50)	χ^2^ = 2.65; *p* = 0.266
	AG	18 (46.15)	5 (23.81)		2 (28.57)	3 (37.50)	
	GG	9 (23.08)	6 (28.57)		0	2 (25.00)	
	A	42 (53.85)	25 (59.52)	p_F_ = 0.570	12 (85.71)	9 (56.25)	p_F_ = 0.118
	G	36 (46.15)	17 (40.48)		2 (14.29)	7 (43.75)	
CASP3 rs113420705	TT	3 (7.69)	3 (14.29)	χ^2^ = 2.05; *p* = 0.358	4 (57.14)	3 (37.50)	χ^2^ = 0.75; *p* = 0.687
	TC	27 (69.23)	16 (76.19)		2 (28.57)	4 (50.00)	
	CC	9 (23.08)	2 (9.52)		1 (14.29)	1 (12.50)	
	T	33 (42.31)	22 (52.38)	p_F_ = 0.339	10 (71.43)	10 (62.50)	p_F_ = 0.709
	C	45 (57.69)	20 (47.62)		4 (28.57)	6 (37.50)	

Abbreviations: χ^2^—chi-squared test; *p*-value—level of significance. *—statistically significant result.

**Table 6:** Dominant, recessive and overdominant models for IFNAR2 rs2236757.

Model	Genotype	Mild/ Moderate COVID-19	Severe COVID-19/MIS-C	Control	χ^2^, *p*-Value	OR (95% CI)	*p*-Value for OR
Dominant	GG	10 (33.33)	8 (26.67)	7 (46.67)	χ^2^ = 1.80 *p* = 0.406	0.42 (0.11–1.52)	0.185
	GA-AA	20 (66.67)	22 (73.33)	8 (53.33)		2.41 (0.66–8.81)	
Recessive	GG-GA	21 (70.00)	18 (60.00)	13 (86.67)	χ^2^ = 3.54 *p* = 0.187	0.23 (0.04–1.21)	0.083
	AA	9 (30.00)	12 (40.00)	2 (13.33)		4.33 (0.83–22.75)	
Overdominant	GG-AA	19 (63.33)	20 (66.67)	9 (60.00)	χ^2^ = 0.20 *p* = 0.904	1.33 (0.37–4.80)	0.660
	GA	11 (36.67)	10 (33.33)	6 (40.00)		0.75 (0.21–2.70)	

Abbreviations: χ^2^—chi-squared test; *p*-value—level of significance; OR—odds ratio (calculated for the outcome “Severe COVID-19/MIS-C”); 95% CI—95% confidence interval (severe COVID-19/MIS-C—outcome in OR calculation).

**Table 7:** Dominant, recessive and overdominant models for TYK2 rs2304256.

Model	Genotype	Mild/ Moderate COVID-19	Severe COVID-19/MIS-C	Control	χ^2^, *p*-Value	OR (95% CI)	*p*-Value for OR
Dominant	CC	17 (56.67)	11 (36.67)	9 (60.00)	χ^2^ = 3.26 *p* = 0.197	0.39 (0.11–1.38)	0.143
	CA−AA	13 (43.33)	19 (63.33)	6 (40.00)		2.59 (0.73–9.25)	
Recessive	CC−CA	27 (90.00)	24 (80.00)	14 (93.33)	χ^2^ = 2.02 *p* = 0.364	0.29 (0.03–2.62)	0.268
	AA	3 (10.00)	6 (20.00)	1 (6.67)		3.50 (0.38–32.14)	
Overdominant	CC−AA	20 (66.67)	17 (56.67)	10 (66.67)	χ^2^ = 0.77 *p* = 0.681	0.65 (0.18–2.38)	0.520
	CA	10 (33.33)	13 (43.33)	5 (33.33)		1.53 (0.42–5.58)	

Abbreviations: χ^2^—chi-squared test; *p*-value—level of significance; OR—odds ratio (calculated for the outcome “Severe COVID-19/MIS-C”); 95% CI—95% confidence interval (severe COVID-19/MIS-C—outcome in OR calculation).

**Table 8:** Dominant, recessive and overdominant models for OAS1 (rs10774671).

Model	Genotype	Mild/ Moderate COVID-19	Severe COVID-19/MIS-C	Control	χ^2^, *p*-Value	OR (95% CI)	*p*-Value for OR
Dominant	GG	8 (26.67)	5 (16.67)	8 (53.33)	χ^2^ = 6.71 *p* = 0.035 *	0.18 (0.04–0.71)	0.015 *
	GA−AA	22 (73.33)	25 (83.33)	7 (46.67)		5.71 (1.41–23.10)	
Recessive	GG−GA	19 (63.33)	20 (66.67)	15 (100.00)	χ^2^ = 7.37 *p* = 0.025 *	0.06 (0.01–1.16)	0.063
	AA	11 (36.67)	10 (33.33)	0		15.88 (0.86–292.28)	
Overdominant	GG−AA	19 (63.33)	15 (50.00)	8 (53.33)	χ^2^ = 1.14 *p* = 0.567	0.88 (0.25–3.03)	0.833
	GA	11 (36.67)	15 (50.00)	7 (46.67)		1.14 (0.33–3.96)	

Abbreviations: χ^2^—chi-squared test; *p*-value—level of significance; OR—odds ratio (calculated for the outcome “Severe COVID-19/MIS-C”); 95% CI—95% confidence interval (severe COVID-19/MIS-C—outcome in OR calculation). *—statistically significant result.

**Table 9:** Dominant, recessive and overdominant models for OAS3 rs10735079.

Model	Genotype	Mild/ Moderate COVID-19	Severe COVID-19/MIS-C	Control	χ^2^, *p*-Value	OR (95% CI)	*p*-Value for OR
Dominant	AA	16 (53.33)	12 (40.00)	10 (66.67)	χ^2^ = 2.99 *p* = 0.225	0.33 (0.09–1.22)	0.098
	AG−GG	14 (46.67)	18 (60.00)	5 (33.33)		3.00 (0.82–10.99)	
Recessive	AA−AG	24 (80.00)	22 (73.33)	13 (86.67)	χ^2^ = 1.11 *p* = 0.573	0.42 (0.08–2.30)	0.320
	GG	6 (20.00)	8 (26.67)	2 (13.33)		2.36 (0.43–12.87)	
Overdominant	AA−GG	22 (73.33)	20 (66.67)	12 (80.00)	χ^2^ = 0.93 *p* = 0.629	0.50 (0.11–2.19)	0.357
	AG	8 (26.67)	10 (33.33)	3 (20.00)		2.00 (0.46–8.75)	

Abbreviations: χ^2^—chi-squared test; *p*-value—level of significance; OR—odds ratio (calculated for the outcome “Severe COVID-19/MIS-C”); 95% CI—95% confidence interval (severe COVID-19/MIS-C—outcome in OR calculation).

**Table 10:** Dominant, recessive and overdominant models for CD40 rs4813003.

Model	Genotype	Mild/ Moderate COVID-19	Severe COVID-19/MIS-C	Control	χ^2^, *p*-Value	OR (95% CI)	*p*-Value for OR
Dominant	CC	26 (86.67)	27 (90.00)	10 (66.67)	χ^2^ = 4.32 *p* = 0.116	4.50 (0.90–22.40)	0.066
	CT-TT	4 (13.33)	3 (10.00)	5 (33.33)		0.22 (0.04–1.11)	
Recessive	CC-CT	30 (100.00)	30 (100.00)	14 (93.33)	χ^2^ = 4.05 *p* = 0.132	6.31 (0.24–164.57)	0.268
	TT	0	0	1 (6.67)		0.16 (0.01–4.13)	
Overdominant	CC-TT	26 (86.67)	27 (90.00)	11 (73.33)	χ^2^ = 2.29 *p* = 0.318	3.27 (0.63–17.09)	0.160
	CT	4 (13.33)	3 (10.00)	4 (26.67)		0.31 (0.06–1.60)	

Abbreviations: χ^2^—chi-squared test; *p*-value—level of significance; OR—odds ratio (calculated for the outcome “Severe COVID-19/MIS-C”); 95% CI—95% confidence interval (severe COVID-19/MIS-C—outcome in OR calculation).

**Table 11:** Dominant, recessive and overdominant models for FCGR2A rs1801274.

Model	Genotype	Mild/ Moderate COVID-19	Severe COVID-19/MIS-C	Control	χ^2^, *p*-Value	OR (95% CI)	*p*-Value for OR
Dominant	AA	12 (40.00)	10 (33.33)	8 (53.33)	χ^2^ = 1.67 *p* = 0.435	0.44 (0.12–1.55)	0.201
	AG + GG	18 (60.00)	20 (66.67)	7 (46.67)		2.29 (0.64–8.11)	
Recessive	AA + AG	24 (80.00)	21 (70.00)	13 (86.67)	χ^2^ = 1.79 *p* = 0.409	0.36 (0.07–1.93)	0.232
	GG	6 (20.00)	9 (30.00)	2 (13.33)		2.79 (0.52–14.96)	
Overdominant	AA + GG	18 (60.00)	19 (63.33)	10 (66.67)	χ^2^ = 0.20 *p* = 0.905	0.86 (0.23–3.19)	0.826
	AG	12 (40.00)	11 (36.67)	5 (33.33)		1.16 (0.31–4.27)	

Abbreviations: χ^2^—chi-squared test; *p*-value—level of significance; OR—odds ratio (calculated for the outcome “Severe COVID-19/MIS-C”); 95% CI—95% confidence interval (severe COVID-19/MIS-C—outcome in OR calculation).

**Table 12:** Dominant, recessive and overdominant models for CASP3 rs113420705.

Model	Genotype	Mild/ Moderate COVID-19	Severe COVID-19/MIS-C	Control	χ^2^, *p*-Value	OR (95% CI)	*p*-Value for OR
Dominant	TT	3 (10.00)	3 (10.00)	7 (46.67)	χ^2^ = 11.26 *p* = 0.004 *	0.13 (0.03–0.61)	0.010 *
	TC + CC	27 (90.00)	27 (90.00)	8 (53.33)		7.88 (1.65–37.69)	
Recessive	TT + TC	25 (83.33)	24 (80.00)	13 (86.67)	χ^2^ = 0.33 *p* = 0.850	0.62 (0.11–3.50)	0.584
	CC	5 (16.67)	6 (20.00)	2 (13.33)		1.63 (0.29–9.23)	
Overdominant	TT + CC	8 (26.67)	9 (30.00)	9 (30.00)	χ^2^ = 5.39 *p* = 0.068	0.29 (0.08–1.04)	0.058
	TC	22 (73.33)	21 (70.00)	6 (40.00)		3.50 (0.96–12.78)	

Abbreviations: χ^2^—chi-squared test; *p*-value—level of significance; OR—odds ratio (calculated for the outcome “Severe COVID-19/MIS-C”); 95% CI—95% confidence interval (severe COVID-19/MIS-C—outcome in OR calculation). *—statistically significant result.

**Table 13:** Association of risk alleles in children with COVID-19 and healthy control.

Combination of Risk Alleles	COVID-19	Control	*p*	χ^2^, *p*
1 risk alleles	0	0	*p* < 0.001 *	χ^2^ = 24.40; *p* < 0.001 *
2 risk alleles	0	2 (13.33)		
3 risk alleles	1 (1.67)	4 (26.67)		
4 risk alleles	10 (16.67)	3 (20.00)	*p* = 0.750	
5 risk alleles	14 (23.33)	2 (13.33)		
6 risk alleles	17 (28.33)	4 (26.67)		
7 risk alleles	14 (23.33)	0	*p* = 0.034 *	
8 risk alleles	4 (6.67)	0		

Abbreviations: χ^2^—chi-squared test; *p*-value—level of significance. *—statistically significant result.

**Table 14:** Logistic regression analysis for COVID-19 susceptibility prediction in the pediatric population.

Variable	B	S.E.	Wald	df	*p*	Exp (B)	95% CI for Exp (B)	
							Lower	Upper
Constant	4.33	1.28	11.44	1	0.001 *	75.77		
Sex Male/Female	1.01	0.53	3.63	1	0.057	2.73	0.97	7.70
ACE2 rs2074192 C/T	−1.05	0.54	3.73	1	0.053	0.35	0.12	1.02
IFNAR2 rs2236757 G/A	−1.19	0.58	4.15	1	0.042 *	0.31	0.10	0.96
TYK2 rs2304256 C/A	−0.32	0.63	0.26	1	0.611	0.73	0.21	2.51
OAS1 rs10774671 G/A	−2.14	0.62	12.02	1	0.001 *	0.12	0.04	0.40
OAS3 rs10735079 A/G	−1.26	0.63	4.00	1	0.046 *	0.28	0.08	0.98
CD40 rs4813003 C/T	1.99	0.82	5.93	1	0.015 *	7.30	1.47	36.14
FCGR2A rs1801274 A/G	−0.40	0.55	0.51	1	0.475	0.67	0.23	1.99
CASP3 rs113420705 T/C	−2.33	0.64	13.11	1	<0.001 *	0.10	0.03	0.34

Abbreviations: B—predictor’s logistic coefficient; S.E.—standard error for B; Wald—Wald chi-square value; df—degree of freedom; *p*—level of statistical significance; Exp (B)—odds ratio for indicators—sex “Male” and dominant gene’s allele; 95% CI for Exp (B)—95% confidence interval for Exp (B). *—statistically significant result.

**Table 15:** Logistic regression analysis for severe COVID-19 course and MIS-C prediction in the pediatric population.

Variable	B	S.E.	Wald	df	*p*	Exp (B)	95% CI for Exp (B)	
							Lower	Upper
Constant	2.54	1.61	2.51	1	0.113	12.71		
Sex Male/Female	2.68	1.05	6.48	1	0.011 *	14.59	1.85	114.80
ACE2 rs2074192 C/T	−2.08	0.86	5.82	1	0.016 *	0.13	0.02	0.68
IFNAR2 rs2236757 G/A	−2.98	1.06	7.86	1	0.005 *	0.05	0.01	0.41
TYK2 rs2304256 C/A	−0.65	0.93	0.49	1	0.486	0.52	0.08	3.24
OAS1 rs10774671 G/A	−2.47	0.98	6.33	1	0.012 *	0.09	0.01	0.58
OAS3 rs10735079 A/G	−1.20	0.82	2.15	1	0.143	0.30	0.06	1.50
CD40 rs4813003 C/T	5.58	1.93	8.36	1	0.004 *	264.57	6.03	11,601.05
FCGR2A rs1801274 A/G	−1.19	0.89	1.78	1	0.182	0.31	0.05	1.74
CASP3 rs113420705 T/C	−3.72	1.11	11.31	1	0.001 *	0.02	0.01	0.21

Abbreviations: B—predictor’s logistic coefficient; S.E.—standard error for B; Wald—Wald chi-square value; df—degree of freedom; *p*—level of statistical significance; Exp (B)—odds ratio for indicators—sex “Male” and dominant gene’s allele; 95% CI for Exp (B)—95% confidence interval for Exp (B). *—statistically significant result.

**Table 16:** Gene network interactions.

Co-Expression	Physical Interactions	Shared Protein Domains	Co-Localization	Predicted Functional Relationships
ACE2—ACE	ACE2—ACE	ACE2—ACE	ACE—CFLAR	ACE2—AGT
ACE2—CLTRN	ACE2—AGT	ACE2—CLTRN	IFNAR2—IL12RB1	ACE2—GHRL
IFNAR2—TRAF3	AGT—ACE	IFNAR2—IL12RB1	IFNAR2—RACK1	IFNAR2—JAK1
OAS1—OAS3	IFNAR2—TYK2	TYK2—JAK1	IFNAR2—TRAF2	IFNAR2—TYK2
OAS1—OAS2	IFNAR2—JAK1	OAS1—OAS3	OAS1—OAS3	TYK2—JAK1
OAS1—OASL	IFNAR2—RACK1	OAS1—OAS2	OAS1—OAS2	IFNAR2—RACK1
OAS1—FCGR2A	TYK2—JAK1	OAS1—OASL	OAS3—OAS2	OAS1—OASL
OAS2—OAS3	TYK2—RACK1	OAS2—OASL	JAK1—CFLAR	OAS3—OAS2
OAS2—OASL	TYK2—IL12RB1	OAS3—OASL		OAS2—OASL
OAS2—IFNAR2	TYK2—JAKMIP1	OAS3—OAS2		FCGR2A—GP6
OAS2—NEO1	JAKMIP1—JAK1	OAS1—TENT4B		CD40—TRAF2
OAS2—FCGR2A	RACK1—JAK1	OAS2—TENT4B		CD40—TXN
FCGR2A—CFLAR	CD40—TRAF2	OAS3—TENT4B		CASP3—TRAF3
FCGR2A—CD40	CD40—TRAF3	OAS1—PAPOLG		TRAF3—CFLAR
CD40—JAK1	CASP3—NDUFS1	OAS2—PAPOLG		
CD40—TXN	CASP3—NEO1	OAS3—PAPOLG		
CD40—OAS1	CASP3—TXN	OAS1—PAPOLB		
CD40—OAS3	CASP3—CFLAR	OAS2—PAPOLB		
CD40—CFLAR	CFLAR—TRAF2	OAS3—PAPOLB		
CFLAR—JAK1		PAPOLG—TENT4B		
CFLAR—TXN		PAPOLB—TENT4B		
CFLAR—TRAF2		PAPOLB—PAPOLG		
		FCGR2A—GP6		
		CASP3—CFLAR		
		TRAF2—TRAF3		

## Data Availability

Data are contained within the article and Supplementary Material.

## Supplementary Materials

The following supporting information can be downloaded at: https://www.mdpi.com/article/10.3390/v15102093/s1, Table S1: Test for Hardy–Weinberg equilibrium for ACE2 rs2074192, IFNAR2 rs2236757 and TYK2 rs2304256. Table S2: Test for Hardy–Weinberg equilibrium for OAS1 rs10774671 and OAS3 rs10735079. Table S3: Test for Hardy–Weinberg equilibrium for CD40 rs4813003, FCGR2A rs1801274 and CASP3 rs113420705.

## Limitations of the Study

Study limitations—small sample size (*n* = 75). It was a pilot study with a fixed effect size of 0.5.
