# Supplementary material for: The Relationship between COVID-19 Severity in Children and Immunoregulatory Gene Polymorphism
Source: Viruses. 2023 Oct 14;15(10):2093. doi: 10.3390/v15102093 (PMC10612096; doi:10.3390/v15102093)
Supplement: Supplementary file 1 [file viruses-15-02093-s001.zip › viruses-2628787-supplementary.pdf]

Supplementary Materials: The following supporting information can be downloaded at: https://www.mdpi.com/article/10.3390/v15102093/s1, Table S1: Test for Hardy–Weinberg equilibrium for ACE2 rs2074192, IFNAR2 rs2236757 and TYK2 rs2304256. Table S2: Test for Hardy–Weinberg equilibrium for OAS1 rs10774671 and OAS3 rs10735079. Table S3: Test for Hardy–Weinberg equilibrium for CD40 rs4813003, FCGR2A rs1801274 and CASP3 rs113420705.

Table S1. Test for Hardy-Weinberg equilibrium for ACE2 rs2074192, IFNAR2 rs2236757, TYK2 rs2304256

Group	Genotype	ACE2 rs2074192		IFNAR2 rs2236757		TYK2 rs2304256	
		Expected	Observed	Expected	Observed	Expected	Observed
All patients	Common Homozygotes	19.3	29	19.8	25	34.7	37
	Heterozygotes	37.5	17	37.5	27	32.6	28
	Rare Homozygotes	18.3	29	17.8	23	7.7	10
	χ^2, p-value	$\chi^2 = 22.41$; $p < 0.001$*		$\chi^2 = 5.86$; $p = 0.016$*		$\chi^2 = 1.52$; $p = 0.218$	
COVID-19 patients	Common Homozygotes	12.6	21	13.5	18	26.0	28
	Heterozygotes	29.8	13	29.9	21	27.0	23
	Rare Homozygotes	17.6	26	16.5	21	7.0	9
	χ^2, p-value	$\chi^2 = 19.06$; $p < 0.001$*		$\chi^2 = 5.34$; $p = 0.021$*		$\chi^2 = 1.31$; $p = 0.252$	
Control group (noninfected persons)	Common Homozygotes	6.67	8	6.7	7	8.8	9
	Heterozygotes	6.67	4	6.7	6	5.4	5
	Rare Homozygotes	1.67	3	1.7	2	0.8	1
	χ^2, p-value	$\chi^2 = 2.40$; $p = 0.121$		$\chi^2 = 0.15$; $p = 0.696$		$\chi^2 = 0.07$; $p = 0.791$	

Abbreviations: χ^2 – chi-squared test, p-value – level of its significance.
* – statistically significant result.

Table S2. Test for Hardy-Weinberg equilibrium for OAS1 rs10774671, OAS3 rs10735079

Group	Genotype	OAS1 rs10774671)		OAS3 rs10735079	
		Expected	Observed	Expected	Observed
All patients	Common Homozygotes	19.5	21	31.4	38
	Heterozygotes	38.0	33	34.3	21
	Rare Homozygotes	18.5	21	9.4	16
	χ^2, p-value	$\chi^2 = 1.31$; $p = 0.299$		$\chi^2 = 11.25$; $p < 0.001$*	
COVID-19 patients	Common Homozygotes	11.3	13	22.8	28
	Heterozygotes	29.5	26	28.4	18
	Rare Homozygotes	19.3	21	8.8	14
	χ^2, p-value	$\chi^2 = 0.83$; $p = 0.362$		$\chi^2 = 8.01$; $p = 0.005$*	
Control group (noninfected persons)	Common Homozygotes	8.8	8	8.8	10
	Heterozygotes	5.4	7	5.4	3
	Rare Homozygotes	0.8	0	0.8	2
	χ^2, p-value	$\chi^2 = 1.39$; $p = 0.238$		$\chi^2 = 2.92$; $p = 0.088$	

Abbreviations: χ^2 – chi-squared test, p-value – level of its significance.
* – statistically significant result.

Table S3. Test for Hardy-Weinberg equilibrium for CD40 rs4813003, FCGR2A rs1801274, CASP3 rs113420705

**Group**	**Genotype**	**CD40 rs4813003**		**FCGR2A rs1801274**		**CASP3 rs113420705**	
		**Expected**	**Observed**	**Expected**	**Observed**	**Expected**	**Observed**
All patients	Common Homozygotes	62.6	63	25.8	30	18.8	13
	Heterozygotes	11.9	11	36.4	28	37.5	49
	Rare Homozygotes	0.6	1	12.8	17	18.8	13
	χ^2, p-value	$\chi^2 = 0.41$; $p = 0.230$		$\chi^2 = 3.97$; $p = 0.046$*		$\chi^2 = 7.05$; $p = 0.008$*	
COVID-19 patients	Common Homozygotes	53.2	53	18.7	22	12.6	6
	Heterozygotes	6.6	7	29.6	23	29.8	43
	Rare Homozygotes	0.2	0	11.7	15	17.6	11
	χ^2, p-value	$\chi^2 = 0.63$; $p = 0.230$		$\chi^2 = 2.98$; $p = 0.084$		$\chi^2 = 11.79$; $p < 0.001$*	
Control group (noninfected persons)	Common Homozygotes	9.6	10	7.4	8	6.7	7
	Heterozygotes	4.8	4	6.3	5	6.7	6
	Rare Homozygotes	0.6	1	1.4	2	1.7	2
	χ^2, p-value	$\chi^2 = 0.42$; $p = 0.519$		$\chi^2 = 0.64$; $p = 0.424$		$\chi^2 = 0.15$; $p = 0.699$	

Abbreviations: χ^2 – chi-squared test, p-value – level of its significance.

* – statistically significant result.
